# Nanocarrier-Based Delivery of SN22 as a Tocopheryl Oxamate Prodrug Achieves Rapid Tumor Regression and Extends Survival in High-Risk Neuroblastoma Models

**DOI:** 10.3390/ijms23031752

**Published:** 2022-02-03

**Authors:** Ivan S. Alferiev, David T. Guerrero, Danielle Soberman, Peng Guan, Ferro Nguyen, Venkatadri Kolla, Ilia Fishbein, Blake B. Pressly, Garrett M. Brodeur, Michael Chorny

**Affiliations:** Department of Pediatrics, Children’s Hospital of Philadelphia, Perelman School of Medicine, The University of Pennsylvania, Philadelphia, PA 19104, USA; alferiev@chop.edu (I.S.A.); david.travis.guerrero@gmail.com (D.T.G.); danielle.soberman@gmail.com (D.S.); guanp@chop.edu (P.G.); nguyenf@chop.edu (F.N.); kolla@chop.edu (V.K.); fishbein@chop.edu (I.F.); presslyb@chop.edu (B.B.P.); brodeur@chop.edu (G.M.B.)

**Keywords:** neuroblastoma, drug resistance, topoisomerase I inhibitor, SN22, high-risk disease, nanoparticle, prodrug, mitocan, orthotopic xenograft model, bioluminescent imaging

## Abstract

Despite the use of intensive multimodality therapy, the majority of high-risk neuroblastoma (NB) patients do not survive. Without significant improvements in delivery strategies, anticancer agents used as a first-line treatment for high-risk tumors often fail to provide clinically meaningful results in the settings of disseminated, recurrent, or refractory disease. By enhancing pharmacological selectivity, favorably shifting biodistribution, strengthening tumor cell killing potency, and overcoming drug resistance, nanocarrier-mediated delivery of topoisomerase I inhibitors of the camptothecin family has the potential to dramatically improve treatment efficacy and minimize side effects. In this study, a structurally enhanced camptothecin analog, SN22, reversibly coupled with a redox-silent tocol derivative (tocopheryl oxamate) to allow its optimally stable encapsulation and controlled release from PEGylated sub-100 nm nanoparticles (NP), exhibited strong NB cell growth inhibitory activity, translating into rapid regression and durably suppressed regrowth of orthotopic, *MYCN*-amplified NB tumors. The robust antitumor effects and markedly extended survival achieved in preclinical models recapitulating different phases of high-risk disease (at diagnosis vs. at relapse with an acquired loss of p53 function after intensive multiagent chemotherapy) demonstrate remarkable potential of SN22 delivered in the form of a hydrolytically cleavable superhydrophobic prodrug encapsulated in biodegradable nanocarriers as an experimental strategy for treating refractory solid tumors in high-risk cancer patients.

## 1. Introduction

Neuroblastoma (NB), a neural crest-derived malignancy of the sympathetic nervous system, is the most common and deadly extracranial solid tumor of childhood. It accounts for 6–10% of all childhood cancers, and 12–15% of deaths from cancer in children [1]. Despite major improvements in the cure rates for other pediatric cancers, little progress has been made in patients with aggressive forms of NB. Many high-risk patients have advanced, metastatic tumors at diagnosis, and their disease often progresses relentlessly, demonstrating no durable response to treatment. The intensive multimodality therapy currently used in the clinic ultimately fails in over half of aggressive disease cases: 50–60% experience a relapse with no curative rescue treatment options [2,3]. Amplification of the *MYCN* oncogene encoding a transcription factor N-Myc is present in 18–20% of all NBs [4], and is a high-risk feature predicting low chemosensitivity and poor therapeutic response [5]. A *MYCN*-dependent oncogenic pathway plays a key role in promoting the aggressive, intrinsically resistant disease phenotype [6], in part by modulating the expression of ATP-binding cassette transporters driving active efflux of chemotherapeutics from tumor cells [7]. Among other members of this exporter family representing downstream targets of *MYCN*, ABCG2 is unique in its strong association with a cancer stem cell-enriched tumor phenotype exhibiting inherently high resistance to chemotherapeutic agents [8]. While ABCG2 was demonstrated to be present in all NB cell subtypes [9], its increased expression levels in NB tumors at diagnosis are an established adverse prognostic factor [10]. Susceptibility to ABCG2-driven efflux severely compromises the performance of different classes of chemotherapeutics, including topoisomerase I inhibitors of the camptothecin family clinically used as a first-line treatment for NB [11,12,13,14]. In this context, nanocarrier-based delivery of SN22, a camptothecin analog protected from ABCG2-mediated export and enzymatic inactivation, may offer a significant therapeutic benefit by combating drug resistance in the *MYCN*-driven aggressive disease.

SN22 is a topoisomerase I inhibitor with potent anticancer activity demonstrated in early preclinical studies [15,16,17]. However, due to its lack of solubility in standard delivery vehicles, its hydroxylated analog (SN38), amenable to hydrophilic modifications, was chosen as the starting material for making a water-soluble carbamate derivative, irinotecan, later implemented clinically [17,18]. Irinotecan itself is pharmacologically inactive, and its conversion to SN38 takes place primarily in the liver [19,20] through an enzymatic process with low but variable rates, typically not exceeding 3–4% [21]. Besides poor bioavailability due to inefficient recovery from the precursor, it was later discovered that the 10-hydroxy substituent introduced into the structure of SN38 increased its affinity toward ABCG2 [11,22], likely contributing to the unremarkable results observed with irinotecan in preclinical models of immature neuroblastoma with *MYCN* amplification [23,24] and to a lack of efficacy when tested in children with aggressive disease [25]. More recently, a search for camptothecin analogs not susceptible to this resistance mechanism led to a “rediscovery” of SN22 as a specific topoisomerase I inhibitor with potency similar or greater than that of SN38, yet two orders of magnitude less prone to ABCG2-mediated efflux [22,26,27].

Besides addressing the solubility issue and allowing its convenient dosing, SN22 formulation and delivery in biodegradable nanocarriers can markedly improve efficacy and safety of its use by (i) enabling control over the drug biodistribution through adjustments in the nanocarrier design, (ii) extending tumor tissue exposure to therapeutically effective drug levels, and (iii) preventing rapid clearance and activity loss by protecting the chemically labile cargo from premature degradation and allowing for its sustained release from the carrier in the tumor tissue. Through improving selectivity and enhancing potency of its tumor cell killing activity, the nanocarrier-mediated delivery of SN22 can potentially achieve robust and lasting on-target effects at lower or less frequently administered doses, thus further protecting healthy tissues from exposure to toxic drug levels.

NP designed for cancer pharmacotherapy should combine the ability to stably entrap their cargo after administration [28] with a uniform and small (sub-100 nm) size in order to maximize their accumulation in the tumor tissue [29]. However, precise control over the drug release rate from carriers sized in the sub-100 nm range poses a challenge, as the resultant increase in the surface area/volume ratio together with the high diffusivity of a small-molecule payload with moderate lipophilicity, such as SN22, will cause rapid drug escape after systemic administration [30]. Preventing premature drug dissociation and stabilizing carrier-cargo association can be accomplished by encapsulating the drug in the form of its hydrophobized precursor (prodrug) with a bulkier molecular structure, as has been shown by our group and by others [31,32,33,34]. However, the rate of the prodrug activation needs to be coordinated with the time scale of NP uptake and retention in order to allow sustained tumor exposure to therapeutically effective levels of the drug in its biologically active form.

In the present study, we designed and characterized hydrophobized prodrugs of SN22 coupled with bulky tocol promoieties via cleavable ester linkages with different hydrolytic labilities controlled through the “electron displacement effect” [35]. Reversibly hydrophobizing and increasing the size of molecular cargoes through pharmacophore conjugation with tocol residues offers an effective tool for improving drug encapsulation stability. In particular, we have shown that this approach could be successfully applied to stably load SN38 derivatives in PEGylated sub-100 nm NP with polylactide (PLA) cores. In these studies, the redox-silent and carboxylated tocols, *tocopheryl succinate* and *tocopheryl oxyacetate*, were used to create hydrolytically activatable prodrugs demonstrating excellent compatibility with the particle-forming polymer and good drug recovery yields when formulated in small-sized NP made of PLA-PEG co-polymer [31,34]. However, whereas coupling SN38 with these moderately acidic tocol derivatives (pKa = 5.6 and 3.6 for tocopheryl succinate and oxyacetate, respectively [36,37]) can provide phenolic ester prodrugs with adequate activation rates, a similar approach cannot be directly applied to the SN22 lacking the phenolic 10-hydroxy group present in SN38 [38]. Attaching these tocol carboxylates to C-20 on the E-ring of the camptothecin pharmacophore, the only site accessible for esterification in SN22, is expected to provide aliphatic conjugates exhibiting much slower activation kinetics, resulting in significantly reduced amounts of the regenerated bioactive drug [31]. We reasoned that the hydrolytic lability of the prodrug could be restored by using a promoiety constructed with a structurally distinct tocol acid contributing a powerful electron withdrawing effect of an α-carbonyl group positioned adjacent to the ester linkage.

Toward this goal, we designed a prodrug of SN22 reversibly hydrophobized using a novel tocopheryl oxamate derivative (SN22-TOx) and evaluated the drug release kinetics, disassembly rate, and the in vitro NB cell growth inhibitory activity of its nanoparticulate formulation. To elucidate the role of the TOx-based prodrug design, these properties were examined in comparison to a structural analog, where SN22 was coupled with tocopheryl oxyacetate as a hydrophobizing promoiety (SN22-TOA). In another series of experiments, we investigated the therapeutic efficacy of the NP-encapsulated SN22-TOx, NP[SN22-TOx], in the settings of *MYCN*-amplified disease, comparing the magnitude and longevity of responses in preclinical models of newly diagnosed and relapsed, multidrug-resistant NB featuring an acquired loss-of-function mutation in the tumor suppressor protein p53. Learning about the impact of the prodrug design on the tumor cell killing activity of nanoencapsulated SN22 and experimentally demonstrating its effectiveness in animal models that are faithfully reproducing key histological and pathological features of aggressive human disease is expected to inform the further development and implementation of an NP/prodrug-based delivery as a clinically viable approach for treating refractory NB and other aggressive solid tumors.

## 2. Results

**Figure 1:**
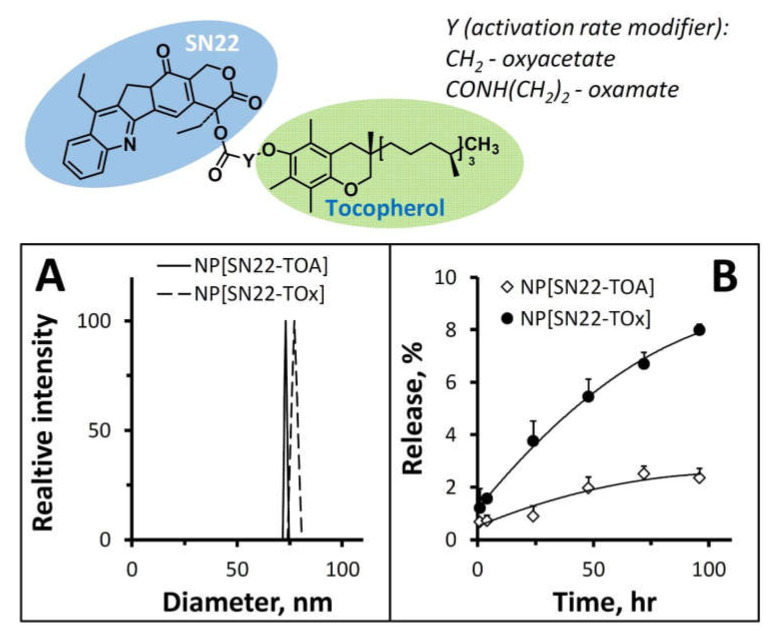
Schematically shown molecular design of SN22-tocol prodrugs with varying hydrolytic labilities and in vitro characterization of their nanoparticle formulations: size distributions (**A**) and release kinetics determined using an external sink method (**B**). Data in (**B**) are presented as mean ± SD.

Tocol-linked prodrugs, SN22-TOx and SN22-TOA (structures shown in Figure 1), were produced by the direct coupling of SN22 to respective tocol acids with 63% and 95% yields, respectively. The predicted organophilicities of both compounds (LogP_octanol/water_ of 9.7 and 9.9, calculated as described in [39]) by far exceed that of the parent SN22 (3.6), allowing their encapsulation in sub-100 nm sized PEGylated NP using a modification of the nanoprecipitation method [40,41]. NP formulations of the structurally analogous and similarly organophilic SN22-TOx and SN22-TOA exhibited comparably high entrapment efficiencies (90–95%, corresponding to a loading of 16–17% *w*/*w*) and a uniform size in the 70–85 nm range (Figure 1A). At the same time, a remarkable difference was observed between their release profiles: after a negligible burst release in the first hour (Figure 1B), the dissociation of SN22 encapsulated, as the rapidly activatable TOx conjugate occurred at a significantly faster rate than that of the less hydrolytically labile TOA-linked prodrug (8.0 ± 0.2% vs. 2.4 ± 0.4% after 96 h, *p* < 0.0001). Remarkably, we observed that, unlike SN22-TOA, which remained largely chemically intact upon the release from NP (76 ± 2% present in the prodrug form), the release medium samples collected from NP[SN22-TOx] contained a significantly more sizeable fraction of regenerated SN22 (43 ± 1%).

**Figure 2:**
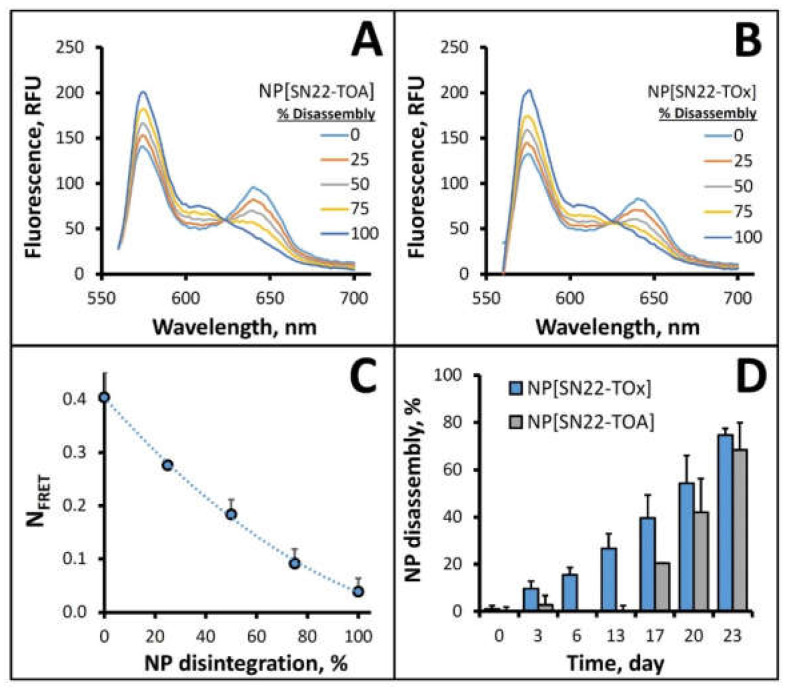
Spectral properties of NP/prodrug formulations and Förster Resonance Energy Transfer studies of NP disassembly. Changes in emission spectra (**A**,**B**) and N_FRET_ (**C**) as a function of the NP integrity status were simulated by combining NP co-labeled with donor and acceptor fluorophores with a 1:1 mixture of singly labeled NP at ratios representing respective disintegration levels. Disassembly of NP/prodrug formulations was monitored fluorimetrically in fetal bovine serum at 37 °C (**D**) based on energy transfer efficiency measurements and the N_FRET_/NP integrity status correlation shown in (**C**). Data in (**C**,**D**) are presented as mean ± SD.

For disassembly kinetics studies, we produced sets of prodrug-loaded NP labeled with the spectrally complementary fluorophores, BODIPY_558/568_ (donor) and BODIPY_650/665_-X (acceptor), either singly or in combination. NP disintegration in serum was monitored in situ using a quantitative assay based on Förster Resonance Energy Transfer (FRET). To model fluorescence patterns at different stages of NP disassembly, we mixed dually and singly labeled NP at different ratios, with 100% dual-labeled NP representing the initial state of full integrity, and a 1:1 mixture of NP singly labeled with the donor or acceptor BODIPY probe simulating the end result of the disintegration process with complete separation of the fluorophores. Similar changes in the emission spectra reflecting progressively declining energy transfer within the donor–acceptor pair and an increase in the donor fluorophore emission at 575 nm upon simulated fluorophore separation were observed regardless of the prodrug design (Figure 2A,B). Accordingly, the scale of these changes, expressed as N_FRET_ [42], was similar for both formulations and ranged from 0.40 ± 0.05 to 0.04 ± 0.02 for simulated NP disassembly degrees of 0% and 100%, respectively (Figure 2C). When this correlation was applied to examine disassembly of the two formulations upon incubation with serum, the prodrug-loaded NP revealed highly dissimilar kinetic patterns that paralleled their different drug release rates: consistent with the faster release of its cargo, the NP[SN22-TOx] formulation also exhibited a relatively high and steady rate of disassembly of ca. 2.9 ± 0.3% per day (*p* < 0.001, Figure 2D). In contrast, NP formulated with SN22-TOA remained initially intact showing no significant disintegration over the first 2 weeks, but quickly caught up over the next 10 days (6.9 ± 0.6% disassembly per day, *p* = 0.007). By day 23, both formulations showed a similar magnitude of integrity loss, with 75 ± 3% and 68 ± 12% disassembly determined for NP[SN22-TOx] and NP[SN22-TOA], respectively, at this time point.

**Figure 3:**
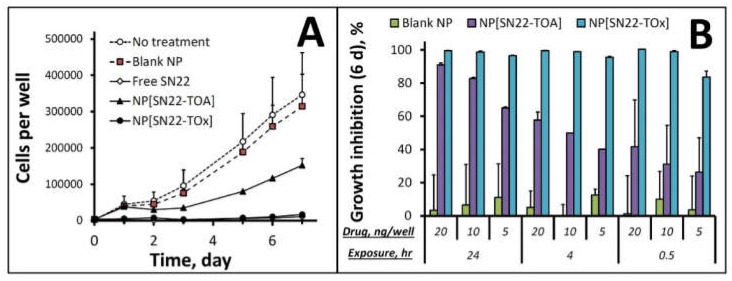
In vitro growth inhibition studies in cultured *MYCN*-amplified neuroblastoma (IMR-32) cells. The effect of NP loaded with SN22 prodrugs (5 ng/well, 24-h exposure) is shown in comparison to ‘no treatment’ or free SN22 and blank NP controls applied to cells at equivalent doses (**A**). The effect of prodrug-loaded NP on IMR-32 cell growth measured at 6 days post-treatment is shown as a function of the drug dose and exposure duration in comparison to blank NP (**B**). Data are presented as mean ± SD.

Consistent with their distinct release and activation rates, the prodrugs formulated in NP exhibited different NB cell growth inhibitory efficiencies: the proliferation of highly malignant, *MYCN*-amplified NB cells derived at diagnosis (IMR-32 [43]) was fully inhibited over 7 days following a 24-h exposure to NP[SN22-TOx] at a low dose equivalent to 5 ng SN22 per well, with potency similar to that of free SN22. In contrast, NP[SN22-TOA] had only a limited tumor cell growth inhibitory effect under these conditions (Figure 3A). Furthermore, whereas the NB cell killing activity of NP-encapsulated SN22-TOx was highly robust at all tested concentrations, even at the shortest exposure of 30 min, the less rapidly activatable SN22-TOA required 24 h of continuous exposure to drug concentrations greater than 10 ng SN22 per well (>265 nM) in order to durably suppress the growth of IMR-32 cells (Figure 3B).

**Figure 4:**
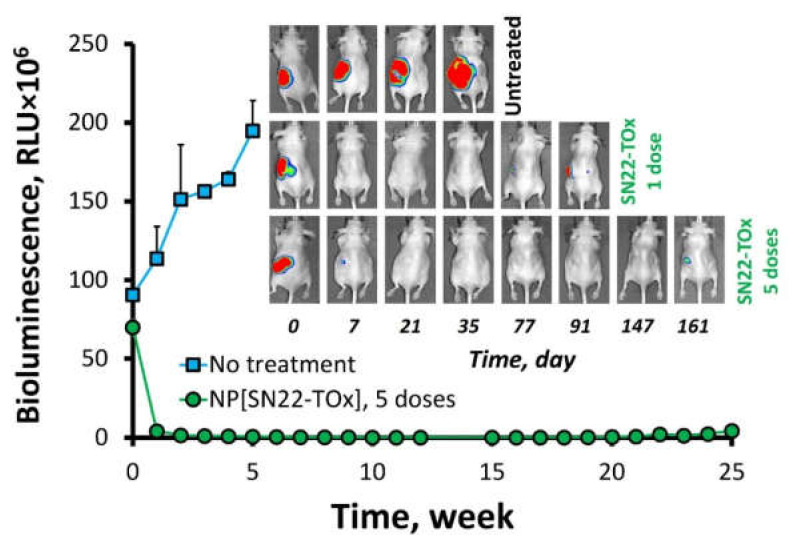
Therapeutic efficacy of the NP-encapsulated SN22-tocopheryl oxamate prodrug in an orthotopic xenograft model of the *MYCN*-amplified, newly diagnosed NB. Mice bearing xenografts established using luciferase-expressing IMR-32 cells were administered with one of five weekly NP doses equivalent to 10 mg SN22 per kg. Tumor growth was continuously monitored by bioluminescent imaging over the course of treatment and after treatment cessation. Data are presented as mean ± SD.

Therapeutic efficacy of SN22 encapsulated in NP, as the tocopheryl oxamate-linked prodrug, was next tested in an orthotopic model of the *MYCN*-amplified, newly diagnosed disease established using IMR-32 cells stably expressing luciferase. The tumor-associated signal rapidly decreased in animals receiving either a single dose or five weekly doses of systemically administered NP[SN22-TOx] and remained uniformly below the detection threshold up to 8 and 20 weeks after treatment cessation, respectively (Figure 4). While a markedly extended survival was observed after a single dose of the NP-encapsulated prodrug corresponding to 10 mg SN22 per kg, all mice treated weekly over 5 weeks survived beyond 25 weeks without exhibiting any adverse effects, confirming that local levels of the bioactive SN22 sufficient for shrinking and durably suppressing regrowth of chemo-naïve *MYCN*-amplified tumors can be maintained with NP-encapsulated SN22-TOx without causing significant systemic toxicity.

**Figure 5:**
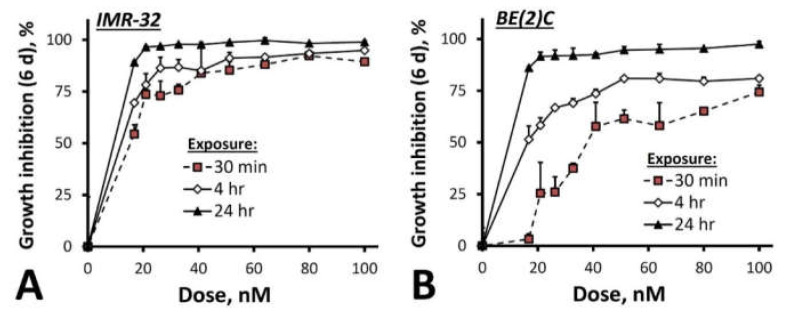
The growth inhibitory effect of SN22 on IMR-32 (**A**) and BE(2)C (**B**) *MYCN*-amplified NB cells derived pre-therapy and at relapse shown at 6 days post-treatment as a function of drug concentration and exposure duration. Cell growth was monitored by bioluminescence using untreated cells as a reference. The weaker response of the BE(2)C cell line in comparison to the chemo-naïve IMR-32 cells reflects a loss of chemosensitivity due to a mutation in the tumor suppressor protein p53 acquired following a course of intensive chemoradiotherapy. Data are presented as mean ± SD.

The response of chemo-naïve IMR-32 cells to SN22 was compared to that of the BE(2)C cell line, which originates from a *MYCN*-amplified NB tumor with a loss-of-function mutation in p53 acquired after a non-curative treatment [44]. Whereas IMR-32 cells were highly sensitive to SN22 at all tested exposure durations (30 min, 4 h and 24 h) with >75% growth inhibition at drug concentrations within the examined 20–100 nM range, BE(2)C cells retained up to 50% of their viability after a 30-min exposure to 40 nM of SN22 (Figure 5B vs. Figure 5A). Four and 24 h of exposure to the drug were required to achieve lasting BE(2)C cell growth inhibition greater than 75% and 90%, respectively.

**Figure 6:**
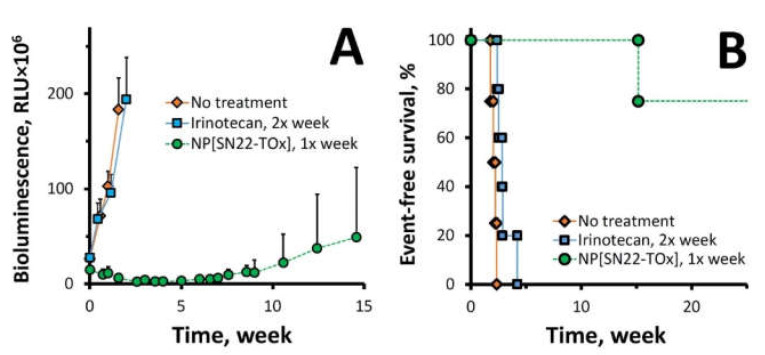
Tumor regression and survival extension in an orthotopic xenograft model of recurrent *MYCN*-amplified NB with acquired chemoresistance in response to the SN22-tocopheryl oxamate prodrug formulated in sub-100 nm PLA-PEG-based NP. Mice orthotopically inoculated with using luciferase-expressing BE(2)C cells were administered with four weekly doses of the NP/prodrug formulation equivalent to 10 mg SN22 per kg, and irinotecan administered twice a week at the equivalent dose (15 mg/kg) was included as a control (5 animals per group). Tumor growth quantitatively determined by bioluminescent imaging (**A**) is shown until the elimination of the first animal in the cohort. Event-free survival was monitored as another therapeutically relevant endpoint (**B**). Data in (**A**) Ftabare presented as mean ± SD.

In agreement with the highly chemoresistant phenotype of the BE(2)C cell line [45], irinotecan administered twice a week at 15 mg/kg was ineffective at inhibiting the growth of BE(2)C xenografts, with all animals reaching the endpoint within less than 5 weeks (Figure 6A,B). In contrast, NP[SN22-TOx] administered weekly over four weeks caused rapid regression of the BE(2)C orthotopic xenografts and markedly reduced the rate of tumor regrowth, extending survival of most animals in this group beyond 26 weeks after the treatment initiation (Figure 6).

## 3. Discussion

NB, with its highly diverse etiology and prevalence of genetically unfavorable variants, poses a need for more efficient treatment strategies, as the intensive, multimodality therapy currently used in the clinic fails to eradicate the disease in over half of high-risk patients [2,3]. Potent, broad-spectrum therapeutics, such as topoisomerase I inhibitors of the camptothecin family currently used in the clinic as a first-line treatment [46], continue to yield poor results in patients with high-risk NB due to rapid clearance, non-specific biodistribution leading to dose-limiting adverse effects [19,20,47], and drug resistance acquired in the course of treatment [8,11]. In relapsed or refractory NB patients, treatment failure was shown to be associated with an increase in threshold drug levels required for effectively suppressing NB cell growth by 1–3 orders of magnitude, reaching values not achievable clinically [48]. Thus, to combat high-risk NB, there is a need for alternative delivery approaches that will enhance tumor localization of a drug and maintain its therapeutically effective levels without increasing systemic exposure, while sensitizing the tumor to the pharmacological effect by suppressing chemoresistance. Our study demonstrates that these requirements can be addressed with NP-mediated delivery of a structurally optimized camptothecin analog, SN22, formulated as a hydrolytically activatable tocopheryl oxamate-linked prodrug.

The tocopheryl oxamate, used here to form a hydrophobic precursor of SN22 suitable for encapsulation in PLA-based NP, is structurally related to a group of redox-silent tocol derivatives (mitocans) previously shown to enhance antitumor effects of different types of chemotherapeutics in vitro and in vivo [49,50,51,52] and to exert strong cytotoxic activity on poorly differentiated NB cells [51]. The most extensively studied examples of mitocans, *tocopheryl succinate* and *tocopheryl oxyacetate*, are carboxylated derivatives of the highly organophilic α-tocopherol [53,54,55]. When incorporated as promoieties in a prodrug molecule, they strengthen the carrier–cargo association by increasing hydrophobicity of the construct to levels sufficient for its stable entrapment in parenterally administered formulations [56]. The choice of the tocol acid is a key element in construction of such in situ activatable prodrugs, as it controls the rate of the ester bond cleavage [35,57] and thus can be used to maximize drug recovery within the target tissue. Although providing a comparable degree of hydrophobization, tocopheryl oxamate is distinct in being an α-carbonyl derivative. This feature allows it to outbalance the relative deficit in the electron-withdrawing effect when coupled with the aliphatic alcohol, SN22, providing otherwise unachievable high rates of prodrug activation. Remarkably, our results suggest that the process of SN22-TOx cleavage may take place concomitantly with dissociation from the carrier and accelerate the release of the payload by breaking the superhydrophobic conjugate into moderately hydrophobic fragments (tocopheryl oxamate and activated SN22), exhibiting significantly lower affinities toward the particle-forming polymer. The faster release of the cargo, making the particle core more accessible to water can, in turn, contribute toward the earlier initiation of the carrier disassembly observed with NP[SN22-TOx]. It is, therefore, likely that payload release from this formulation initially dominated by prodrug cleavage, both promotes and is itself accelerated by the particle matrix decomposition process at later times. This is in contrast to the release of the SN22-TOA rate-limited by the slow diffusion of the uncleaved, superhydrophobic conjugate from intact NP that fully retain their integrity over several days. Thus, the rapidly activatable design of SN22-TOx appears to play a key role by maximizing the recovery of the active SN22 and controlling the drug release from the nanoparticulate formulation.

The importance of the rapidly activatable prodrug construction is evident from the robust killing effect of NP[SN22-TOx] on cultured IMR-32 cells, translating in vivo into rapid regression of IMR-32 orthotopic xenografts and markedly extended survival after five weeks of treatment with the NP/prodrug formulation at a low, non-toxic drug dose. The profound and lasting antitumor effects on IMR-32 cells and xenografts observed with SN22 in our study are in agreement with the relatively high chemosensitivity of *MYCN*-amplified NB cells derived pre-therapy [45,58]. However, a combination of *MYCN* amplification with acquired drug resistance in recurrent NB tumors results in a major shift in responsiveness to several families of chemotherapeutics, including topoisomerase I inhibitors [44,48]. Our in vitro results demonstrate a similar difference in sensitivity toward SN22 between chemo-naïve IMR-32 cells derived at diagnosis and the BE(2)C cell line originating from a NB tumor recurring after intensive multiagent chemotherapy. It is, therefore, remarkable, that the NP[SN22-TOx] administered in four weekly doses to mice bearing orthotopic BE(2)C xenografts achieved rapid regression of the disease and durably suppressed regrowth of the refractory tumors, showing only a marginal response to irinotecan. Notably, the lasting antitumor effects and long-term survival were achieved in this model of recurrent, multidrug-resistant NB integrating several high-risk features (*MYCN* amplification and an acquired loss of p53 function) with low drug doses administered once a week and causing no adverse effects during and after the treatment period.

Tocol mitocans have previously been demonstrated to sensitize NB cells to chemotherapeutics by several mechanisms [49,51], including potent inhibition of *MYCN* expression driving the aggressive clinical behavior and poor therapeutic response in high-risk disease [59]. Thus, applied in combination with other anticancer agents, they can help achieve stronger and more durable response in the settings of *MYCN*-driven high-risk disease. The new redox-silent tocol derivative, tocopheryl oxamate, employed in our study as an element of the prodrug design enabling efficient encapsulation of SN22 and its controlled release from systemically given biodegradable NP, also possesses the defining chemical characteristics of a mitocan [50,60]. Therefore, the likely possibility that TOx and SN22, synchronously regenerated from the common molecular precursor, SN22-TOx, act cooperatively toward greater antitumor effects, warrants further investigation. In summary, the results of the present study demonstrate feasibility and remarkable efficiency of the NP/prodrug delivery strategy applied to the structurally optimized camptothecin analog SN22. Rapid tumor regression and the long-term survival demonstrated in clinically relevant models of pre-therapy and relapsed forms of *MYCN*-amplified disease highlight the potential of NP-encapsulated SN22-TOx as a safe and efficient therapy for high-risk NB and other aggressive solid tumors.

## 4. Materials and Methods

### 4.1. Prodrug Synthesis

To make SN22-TOx, N-(2-D-α-tocopheryloxy)ethyloxamic acid was first prepared in an overall yield of 79% from D-α-tocopherol treated with tetrabutylammonium hydroxide and reacted with bromoacetonitrile in 1-methyl-2-pyrrolidone. The resulting D-α-tocopheryloxyacetonitrile was then reduced to 2-(D-α-tocopheryloxy)ethylamine with lithium aluminium hydride in ethyl ether. The amine was acylated with methyl chlorooxoacetate, forming methyl N-(2-D-α-tocopheryloxy)ethyloxamate, which was subsequently hydrolyzed with water/potassium carbonate to form N-(2-D-α-tocopheryloxy)ethyloxamic acid. Conjugation of this acid to SN22 was carried out by direct coupling in dichloromethane as a solvent, induced by 1-ethyl-3-(3-dimethylaminopropyl)carbodiimide hydrochloride (EDC) and catalyzed by 4-dimethylaminopyridine tosylate (DPTS). The structure and purity of the conjugate were confirmed by ^1^H NMR and TLC (yield: 63%).

SN22-TOA was obtained by acylating SN22 with D-α-tocopheryloxyacetic acid and prepared as previously described [36]. Conjugation was carried out using EDC as a promoter and DPTS as a catalyst. The structure and purity were confirmed by ^1^H NMR and TLC (yield: 95%).

### 4.2. NP[Prodrug] Formulation and Characterization

The prodrug-loaded NP was formulated using a previously reported method adapted for producing uniformly sized, sub-100 nm nanocarriers [16,50]. In brief, 20 mg of Pluronic F-68, 20 mg of the SN22 prodrug constructs, and a total of 100 mg of the particle-forming polymer (poly(D,L-lactide)-*b*-poly(ethylene glycol) with methoxy-terminated poly(ethylene glycol) block, 5000:5000, polydispersity: 1.12, purchased from JenKem Technology USA, Plano, TX, USA) were dissolved in 20 mL of an ethanol/acetone 1:1 mixture. The obtained organic solution was added with stirring to 50 mL of deionized water. The solvents and excess water were removed under gradually reduced pressure. Glucose and hydroxypropyl-β-cyclodextrin (5% *w*/*v* each) were added to adjust tonicity and as cryoprotectants. NP were sterilized by passing through a 0.2 µm membrane (Minisart, Sartorius, Bohemia, NY, USA), lyophilized, stored at −80 °C and reconstituted in deionized water before use. Entrapment yields were determined spectrophotometrically after extraction in *sec*-butanol [16] from baseline-adjusted signals as follows: OD_370_ − (OD_410_ + OD_330_)/2, where OD represents absorbance at an indicated wavelength. NP size was measured by dynamic light scattering and expressed as intensity.

NP disassembly rates were determined using a previously reported in situ approach based on changes in FRET between two spectrally complementary fluorophores initially co-localized in the particle matrix [61]. NP disintegration increases the distance between labeled polymer fragments, thereby reducing the energy transfer efficiency between the donor and acceptor probes (BODIPY_558/568_ and BODIPY_650/665_-X, respectively). This separation results in readily quantifiable changes in normalized FRET efficiency (N_FRET_), a validated parameter developed for global FRET analysis [42,62]. N_FRET_ was calculated as previously reported [61,62] from NP[don/acc] emission intensities at λ_ex_/λ_em_ = 540 nm/640 nm, λ_ex_/λ_em_ = 540 nm/575 nm and λ_ex_/λ_em_ = 590 nm/640 nm ($I_{540/640}^{don/acc}$, $I_{540/575}^{don/acc}$, and $I_{590/640}^{don/acc}$, respectively) using singly labeled NP to obtain channel bleed-through coefficients ($I_{540/640}^{don}$/$I_{540/575}^{don}$ and $I_{540/640}^{acc}$/$I_{590/640}^{acc}$ ratios measured for PLA-BODIPY_558/568_- and PLA-BODIPY_650/665_-X-labeled NP, respectively) shown as *a* and *b* in the equation below:

(1) $$N_{FRET}=\frac{I_{540/640}^{don/acc}-I_{540/575}^{don/acc}\times a-I_{590/640}^{don/acc}\times b}{\sqrt{I_{540/575}^{don/acc}\times I_{590/640}^{don/acc}}}$$

For NP disassembly studies, NP/prodrug formulations were prepared as above with the inclusion of fluorescently labeled polylactide conjugates (PLA-BODIPY_558/568_ and PLA-BODIPY_650/665_-X [61]), 2 mg each, within the total of 100 mg of the particle-forming polymer. Singly labeled particles applied as channel bleed-through controls were prepared with omission of one of the PLA-BODIPY conjugates.

A correlation between N_FRET_ and NP disassembly was first established for each NP/prodrug formulation by combining co-labeled NP with increasing ratios of a 1:1 mixture of singly labeled NP to model progressive dissociation of the FRET pair-forming fluorophores. This correlation was then applied to calculate the integrity status of prodrug-loaded NP incubated in fetal bovine serum (FBS) at 37 °C, based on N_FRET_ values obtained in situ at predetermined time points.

Release kinetics were measured under perfect sink conditions using an external sink method [41,63]. Prodrug-loaded NP were incubated with a chemically inert and water-immiscible acceptor medium (methyl tert-butyl ether and n-heptane, 1:1 *v*/*v*) and the release rates were determined spectrophotometrically by monitoring baseline-adjusted signals [OD_360_ − (OD_410_ + OD_310_)/2] in acceptor medium samples withdrawn at predetermined time points. Acceptor medium samples were additionally analyzed by TLC/densitometry for the presence of regenerated SN22.

### 4.3. Cell Culture Studies

The effect of prodrug-loaded NP and free SN22 on NB cell viability and growth was measured longitudinally as a function of drug concentration and exposure duration using firefly luciferase-expressing, *MYCN*-amplified cell lines, as previously reported [45]. IMR-32 and SK-N-BE(2)C cells purchased from the American Type Culture Collection (Manassas, VA, USA) were seeded at ~3500 cells/well on day −1 on 96-well plates using DMEM or DMEM/F-12 (1:1) supplemented with 10% FBS as the medium for the respective cell lines. At indicated times, cells were carefully washed, and their incubation was continued in fresh medium containing D-luciferin potassium salt (PerkinElmer, Bridgeville, PA, USA) as a substrate (50 μg/mL). Cell bioluminescence was measured daily, and % growth inhibition was calculated and plotted at 6 days post treatment for different drug doses and exposure intervals using untreated cells as a reference.

### 4.4. Therapeutic Efficacy Studies in Preclinical Models of High-Risk NB

Animal studies were performed in accordance with protocols approved by the Institutional Animal Care and Use Committee of the Children’s Hospital of Philadelphia (IAC 20-001140). For orthotopic inoculation, one million of luciferase-expressing IMR-32 or BE(2)C cells suspended in 20 µL of Cultrex Basement Membrane Extract (Trevigen, Gaithersburg, MD, USA) were implanted into the adipose tissue surrounding the adrenal capsule of athymic nude (*nu*/*nu*) mice [64]. Tumor burden was monitored by bioluminescent imaging using a Xenogen IVIS Imaging System (Caliper Life Sciences, Hopkinton, MA, USA). Tumor-bearing animals were intravenously administered with an indicated number of weekly doses of NP[SN22-TOx], corresponding to 10 mg SN22 per kg. Control animals were injected either with saline or with irinotecan (15 mg/kg, 2 × week). The tumor size and body weights of all animals were regularly checked, and changes in tumor-associated bioluminescent signal over time were used to compare tumor progression rates and therapeutic responses.

### 4.5. Statistical Analysis

Release and cell growth data were compared by regression analysis. Experimental data are expressed as mean ± standard deviation. Differences were termed significant at *p* < 0.05.

## Data Availability

All study data are included in the article.
